# Adalimumab (Humira®) Induced Recurrent Peritonsillar Abscess in A Patient Received Three Different Anti-TNF Therapies: A Case Report

**DOI:** 10.1007/s12070-022-03203-0

**Published:** 2022-12-06

**Authors:** Sevilay Vural, Mikail Kuşdoğan, Hasan Burak Kaya, Venhar İkiz, Levent Albayrak

**Affiliations:** 1 Department of Emergency Medicine, University Medical Center Groningen, Hanzeplein 1, 9713 GZ Groningen, The Netherlands; 2 Department of Emergency Medicine, Yozgat Bozok University, Yozgat, Turkey

**Keywords:** Anti-TNF, Adalimumab, Etanercept, Infliximab, Peritonsillar abscess, Deep infection

## Abstract

Anti-tumor necrosis factor agents are widely used in treating ankylosing spondylitis, but they increase the risk of infection by suppressing the immune response. Therefore, physicians should be careful about recurrent infections in patients under anti-tumor necrosis factor agents.

## Introduction

Ankylosing spondylitis (AS) is a chronic inflammatory disease mainly affecting the sacroiliac joints and spine. Tumor necrosis factor alpha (TNF-α) plays an essential role in the pathogenesis of AS and many other inflammatory diseases, which can be used as a target point during the treatment. If the disease activity continues despite the first-line therapies, anti-TNF drugs can be used for AS [1]. The most widely used anti-TNF drugs are infliximab, etanercept, and adalimumab. Peritonsillar abscess (PTA) is the most common deep infection of the head and neck, which generally occurs as a complication of acute tonsillitis. There are no reported numbers in the literature regarding PTA prevalence or recurrence rates in immunosuppressed patients.

## Case Report

**Fig. 1:**
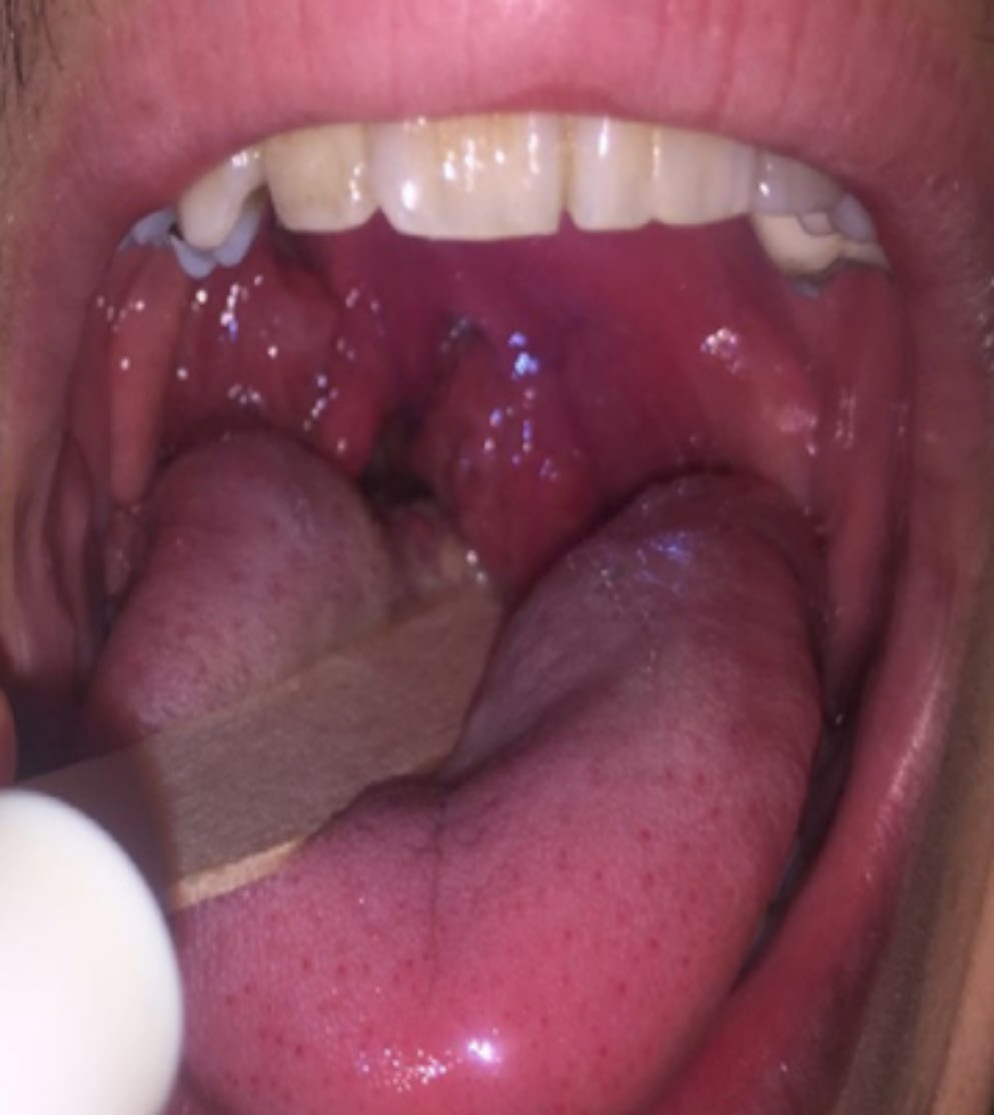
The examination revealing a left-sided peritonsillar abscess without any tonsillitis findings

A 31-year-old non-smoker male patient admitted to the emergency department (ED) with sore throat, dysphagia, and trismus for two days. His vitals were stable.  The physical examination revealed a left-sided peritonsillar abscess without any tonsillitis findings (Fig. 1). His medical history was significant for AS, and receiving anti-TNF treatment for nine years. He had been prescribed etanercept during the first two years and infliximab during the second two-year period. His current treatment was 40 mg Adalimumab (Humira®) for the last five years. It was his third ED admission due to PTA. He had been treated for PTA twice in a different medical center and was hospitalized once during the current year. He denied any similar and/or recurrent medical condition under other anti-TNF therapies. The laboratory tests showed that WBC: 9020 /mm3, neutrophil ratio: 68.2%, and CRP: 9.2 mg/L. The patient was hospitalized by the otolaryngology clinic for further treatment. Abscess drainage was performed, and antibiotic therapy was initiated. No additional pathology such as lymphoma or infectious mononucleosis was found during the differential diagnostics.

Two and a half months after the discharge, he admitted to our ED again with similar complaints. The vitals and laboratory results were similar to the previous admission. He was diagnosed with PTA for the fourth time in two years. The patient was discharged with an antibiotic prescription after the abscess drainage was performed in the ED and referred to his rheumatologist.

## Discussion

 The most common bacterium isolated in PTA are Streptococcus and Fusobacterium, but many abscesses have a mixed profile [2, 3]. Primary treatment strategies are the initiation of appropriate antibiotic therapy and percutaneous evacuation of the abscess if needed. “Recurrence” term for PTA can be defined as a new episode of PTA in ≥ 30 days from the initial PTA [4]. The frequency of recurrent PTA in the normal population is 5.15-16%, and even much higher in younger populations aged 13–18 [5, 6]. As early recurrence can be linked to inadequate treatment, extra-peritonsillar spread of infection, or autoimmune deficiency, late recurrence is more likely related to autoimmune deficiency or prior history of tonsillitis or pharyngitis [6]. Unfortunately, the literature does not answer to the question, “*How many times can a PTA recur.*” Our patient with AS receiving anti-TNF treatment had recurrent PTA attacks (four times), requiring surgical interventions and hospitalizations (two times) in the two-year time period. Although his anti-TNF regimen was the usual suspect, neither the patient himself stated his emergency visits or interventions to his rheumatologist during his follow-up, nor was there any executed rheumatology referral or consultation. Since none of PTAs accompanied by tonsillitis, tonsillectomy was not considered. In addition, the microorganisms causing PTAs were not determined because no culture study was performed. The large-scale studies on the relationship between anti-TNF and infection focused predominantly on tuberculosis activation and opportunistic infections [7, 8]. We could not be sure if PTA episodes were related to any opportunistic microorganism since no culture study was conducted.

As we assume that his repetitive medical condition was associated with the anti-TNF agent, we investigated the drug regimens he received. He had used etanercept and infliximab, and he has been treated with adalimumab for the last five years. All three are the most commonly used anti-TNF agents. He had four PTA during active use of adalimumab. However, the findings comparing the risk of infection in patients treated with etanercept, adalimumab, and infliximab showed no significant difference between the three drugs [9]. In a meta-analysis, significant increases were found in anti-TNF drug use by 20% for any infection, 40% for severe infection, and 250% for tuberculosis reactivation [7]. Nevertheless, we did not encounter any reports or studies including or specifically addressing PTA under anti-TNF treatment.

## Conclusion

 Anti-TNF drugs should also be questioned in specific infection types and repetitive infection patterns that suggest the possibility of immunosuppression in the EDs.

## Abbreviations

AS: Ankylosing spondylitis
TNF-α: Tumor necrosis factor alpha
PTA: Peritonsillar abscess
ED: Emergency Department
